# Organ-Specific Targeted Phenolic Profiling, Antioxidant, and Enzyme-Inhibitory Activities of the Gypsophilous Endemic *Hedysarum pestalozzae*

**DOI:** 10.3390/molecules31132371

**Published:** 2026-07-06

**Authors:** Elif Aktürk Bozdemir

**Affiliations:** Department of Chemistry and Chemical Processing Technologies, Vocational School of Organized Industrial Zone, Tokat Gaziosmanpaşa University, Tokat 60200, Türkiye; elif.akturkbozdemir@gop.edu.tr

**Keywords:** *Hedysarum*, gypsum endemic, LC-ESI-MS/MS, flavonoids, relative antioxidant capacity index (RACI), cholinesterase inhibition, α-glucosidase, Fabaceae

## Abstract

The present study provides an integrative, targeted phenolic–bioactivity mapping of *Hedysarum pestalozzae* Boiss., an endemic Fabaceae species from gypsum habitats of Türkiye. Methanolic extracts of flowers, leaves, stems, and roots were comparatively evaluated for their phenolic profiles, antioxidant potentials, and enzyme inhibitory effects. Targeted LC–ESI–MS/MS analysis of selected phenolics revealed organ-dependent chemical profiles, with flowers exhibiting the highest accumulation of flavonols—hyperoside, kaempferol, quercetin—and phenolic acids such as protocatechuic acid, together with the flavanone glycoside hesperidin, while stems and roots were enriched in catechin, verbascoside, vanillin, and eriodictyol. The antioxidant capacities, assessed through multiple assays (CUPRAC, FRAP, DPPH, ABTS, metal chelation), were integrated using the Relative Antioxidant Capacity Index (RACI), ranking flowers as the most active organ, followed by roots, leaves, and stems. Correlation analysis demonstrated strong positive associations between phenolic abundance and electron-transfer assays, whereas metal chelation showed inverse trends, suggesting distinct antioxidant mechanisms. All extracts exhibited moderate inhibition against cholinesterases, tyrosinase, α-amylase, and α-glucosidase, implying multifunctional enzyme-modulating potential. The root extract showed the highest cholinesterase inhibition, and leaf extract was most effective against α-amylase. These findings indicate that *H. pestalozzae* exhibits organ-dependent chemical diversity and a broad range of biological activities. The present study provides preliminary evidence that *H. pestalozzae* may represent a promising source of bioactive compounds for future phytochemical and biological investigations, warranting further bioassay-guided fractionation, compound isolation, cellular studies, and in vivo validation.

## 1. Introduction

Naturally derived compounds continue to play a leading role in the development of innovative products in pharmaceuticals, nutritional supplements, and dermocosmetics. Phenolics and flavonoids are prominent natural compounds that can both modulate oxidative processes and target specific enzymes. However, the measurement of antioxidant capacity is strongly method-dependent, and no single assay fully captures the biological situation. Current analytical approaches distinguish between reactions based on electron transfer and hydrogen atom transfer and recommend the use of multiplexed measurement panels to capture complementary mechanisms [1,2,3].

The genus *Hedysarum*, a member of the Fabaceae family, comprises approximately 200 species, with a wide geographical distribution extending from Eurasia to North Africa and North America. Recent reviews of the genus highlight the presence of a wide variety of secondary metabolites, such as flavonoids, isoflavonoids, triterpenes/saponins, and coumarins, and their chemotaxonomic importance [4,5]. *Hedysarum* taxa are also notable for their adaptation to gypsum habitats, which present physical and chemical constraints that can influence plant chemistry [6,7].

Gypsic ecosystems serve as natural laboratories for studying plant physiology and metabolism. Gypsophilous species exhibit nutritional signatures and other adaptations reflecting this unusual soil composition (e.g., high calcium and sulfur levels in leaves). It is generally accepted that such edaphic stresses upregulate secondary metabolism and increase the accumulation of defense-related metabolites [7,8]. Türkiye is rich in gypsum landscapes and hosts a high proportion of endemic species; therefore, conducting phytochemical studies on endemic or narrow-range plants specific to gypsum areas is of scientific importance [6].

Despite the increasing number of studies on the genus *Hedysarum* in the literature, there is no comprehensive study integrating organ-based, targeted phenolic analysis and biological activity assays on *Hedysarum pestalozzae* Boiss., a species native to gypsum substrates. In contrast, antioxidant, antimicrobial, and other biological effects have been reported in related species (e.g., *H. coronarium*), and detailed characterizations of different specific metabolites across the genus are reported [4,5,9]. This context supports the likely presence of bioactive phenolic compounds in *H. pestalozzae*. The generation of a core targeted phenolic and biological dataset for this species, along with recently published phylogenetic and cytogenetic studies, will contribute to chemotaxonomic assessments within the subtribe Hedysareae [5].

In our study, antioxidant capacity was measured using several complementary assays. The Relative Antioxidant Capacity Index (RACI), a unit-independent standard score approach, was used to enable comparison across assays with different scales and to rank the samples accordingly. In addition to redox-focused tests, enzyme targets with well-defined clinical and applied relevance were also evaluated: AChE/BChE inhibitors are effective in the management of Alzheimer’s symptoms [10]; inhibition of α-amylase and α-glucosidase slows carbohydrate digestion, limiting postprandial glycemia [11]; and tyrosinase inhibition is a key strategy in the therapeutic and cosmetic management of hyperpigmentation [12].

In this context, we present an organ-level investigation of *H. pestalozzae* based on targeted phenolic analysis and bioactivity assays. (i) Quantification of total phenolic/flavonoid contents and determination of selected phenolics by LC-ESI-MS/MS; (ii) evaluation of antioxidant performance by multiple assays and integration with RACI; (iii) determination of inhibitory activity against AChE, BChE, α-amylase, α-glucosidase, and tyrosinase; and (iv) correlation-based testing of the relationships between chemical composition and biological outcomes are the main components of this study. Combining the ecological context with analytical chemistry and targeted bioassays, this approach aims to place *H. pestalozzae* in its metabolic-functional position within the genus *Hedysarum* and provide primary data for phytochemistry-focused discovery.

## 2. Results and Discussion

### 2.1. Chemical Composition

**Figure 1:**
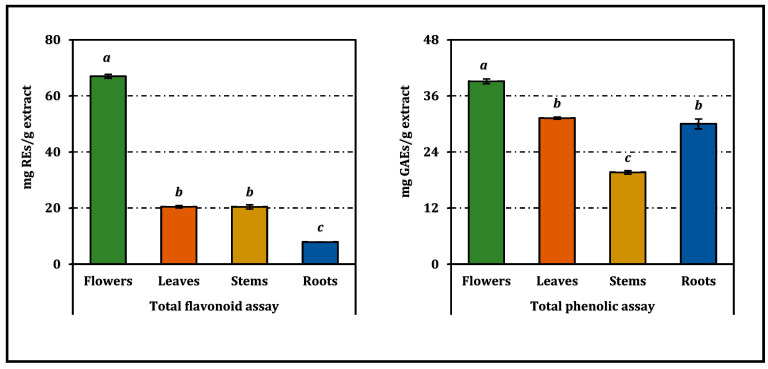
Total flavonoid and phenolic contents of *H. pestalozzae* extracts. REs and GAEs: Rutin and gallic acid equivalents, respectively. Values indicated by the same superscripts (a–c) within the same column are not significantly different according to Tukey’s HSD test at the 5% significance level.

Total phenolics and flavonoids varied markedly among organs (Figure 1). Flowers exhibited the highest total phenolic content (39.14 mg GAEs/g; a), exceeding leaves and roots (31.26 and 30.01 mg GAEs/g) and far surpassing stems (19.65 mg GAEs/g). A similar but even sharper gradient was observed for total flavonoids: flowers accumulated 66.99 mg REs/g, whereas leaves and stems formed an intermediate pair (~20.5 mg REs/g), and roots trailed substantially (7.92 mg REs/g).

Targeted LC analysis resolved a consistent, organ-specific allocation of individual phenolics (Table 1). Flowers were quantitatively dominated by flavonol glycosides and aglycones—hyperoside (6025 ± 28 μg/g), kaempferol (3436 ± 6 μg/g), and quercetin (1916 ± 18 μg/g)—together with high levels of protocatechuic acid (2183 ± 38 μg/g) and hesperidin (1946 ± 13 μg/g). Leaves retained the same constituents but at lower abundance (hyperoside 2750 ± 7 μg/g; kaempferol 2671 ± 39 μg/g; quercetin 509 ± 25 μg/g), while showing relative enrichments of several hydroxycinnamates and phenolic acids, notably chlorogenic (107 ± 1 μg/g), ferulic (330 ± 3 μg/g), and *p*-coumaric acids (152 ± 2 μg/g), and the highest taxifolin among organs (19.2 ± 0.1 μg/g).

Stems contrasted with flowers/leaves by peaking in (+)-catechin (1489 ± 30 μg/g) and verbascoside (339 ± 12 μg/g) and by concentrating luteolin 7-glucoside (72.7 ± 2.7 μg/g), despite comparatively low levels of hyperoside, kaempferol, and quercetin. Roots displayed a distinct, low-flavonoid profile consistent with their minimal total flavonoid content, yet they were uniquely enriched in vanillin (68.1 ± 0.7 μg/g) and showed the highest eriodictyol (13.2 ± 0.5 μg/g) and comparatively elevated syringic acid (72.6 ± 0.9 μg/g). Seven targeted phenolic compounds ((−)-epicatechin, 2-hydroxycinnamic acid, 3,4-dihydroxyphenylacetic acid, apigenin 7-glucoside, apigenin, pinoresinol, and rosmarinic acid) were not detected in any of the analyzed organs and were therefore omitted from Table 1.

Collectively, these data reveal a flower-centric accumulation of flavonols and selected phenolic acids coinciding with the highest total phenolics/total flavonoids, a leaf bias toward chlorogenic/ferulic/*p*-coumaric acids, a stem specialization for catechin/verbascoside/luteolin 7-glucoside, and a root signature marked by vanillin and eriodictyol. These organ-resolved chemotypes align with the global trends in Figure 1 and are supported by compound-wise statistics in Table 1. The chromatographic separation and peak assignment of the identified phenolic compounds are illustrated in Supplementary Figure S2.

The organ-specific phenolic patterns identified in *H. pestalozzae* are consistent with the known chemical framework of the genus, yet present new nuances that expand it. The marked flavonol (hyperoside, quercetin, kaempferol) predominance in flowers reflects the prevalence of these glycosides and aglycones recorded in numerous *Hedysarum* species [13,14,15,16,17,18], while the simultaneous finding of protocatechuic acid is also consistent with previously reported benzoic acids in the genus [19,20]. This pattern may indicate an association between the accumulation of phenolic acids and flavonols, although pathway-level relationships require further validation.

The predominance of hydroxycinnamates (chlorogenic, ferulic, *p*-coumaric) in leaves has previously been associated with structural and photoprotective roles in plants; the presence of long-chain phenylpropanoid esters and related cinnamates in genus compilations supports this capacity [21,22]. Taxifolin is recognized as an intermediate in flavonoid biosynthesis and may reflect differences in secondary metabolite accumulation among organs.

The concentration of (+)-catechin, verbascoside, and luteolin-7-*O*-glucoside in the stem is consistent with catechin patterns reported in *Hedysarum* [23] and may be associated with protective physiological functions in supporting tissues. The prominence of verbascoside may reflect organ-specific accumulation of phenylethanoid compounds [4]. The low flavonoid content, together with elevated vanillin and eriodictyol levels in roots, may reflect organ-specific accumulation patterns of phenylpropanoid-derived metabolites.

The fit of the individual compound distributions with the total phenolics/flavonoids suggests that the observed gradients are not limited to a few peaks but instead reflect coordinated distribution patterns of selected phenolic compounds. The observed decrease in flavonoid abundance along the flower → leaf/stem → root direction is consistent with a decline in flavonol glycosides and their replacement by catechin/phenylethanoid or simpler phenolic acids. Although clear organ-specific differences in phenolic distribution were observed, the underlying biosynthetic and regulatory mechanisms cannot be directly inferred from targeted metabolite profiling alone. Future studies integrating transcriptomic, proteomic, enzymatic, or metabolic flux analyses would provide deeper insights into the regulation of secondary metabolite accumulation in *H. pestalozzae* [24,25].

### 2.2. Antioxidant Activity

**Figure 2:**
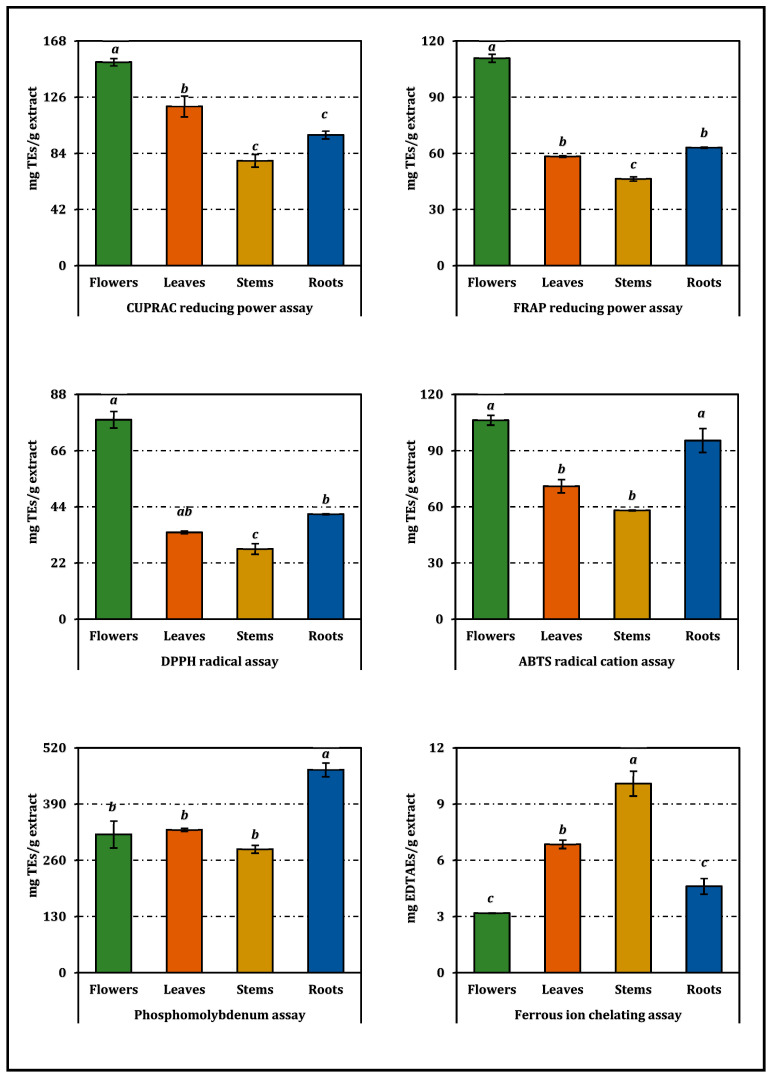
Antioxidant activity of *H. pestalozzae* extracts. TEs and EDTAEs and trolox and ethylenediaminetetraacetic acid (disodium salt) equivalents, respectively. Values indicated by the same superscripts (a–c) on the bar chart are not significantly different according to Tukey’s HSD test at the 5% significance level.

The antioxidant potential of *H. pestalozzae* extracts was evaluated through multiple complementary assays, and the results are summarized in Table 2 and illustrated in Figure 2. Overall, marked variations in activity were observed among different plant parts, reflecting differences in their phenolic and flavonoid contents. The flower extract consistently exhibited the strongest antioxidant capacity across most test systems, while stems showed the weakest responses.

In reducing power assays, the flower extract demonstrated superior efficacy with EC_50_ values of 1.14 mg/mL (CUPRAC) and 0.45 mg/mL (FRAP), indicating a high capacity for electron donation. In contrast, the stem extract displayed the lowest reducing ability, with EC_50_ values of 2.21 mg/mL (CUPRAC) and 1.07 mg/mL (FRAP). Similarly, the flower extract showed the highest radical scavenging potential in both DPPH and ABTS assays (IC_50_ = 3.26 and 1.65 mg/mL, respectively), whereas the stem extract was the least active. The phosphomolybdenum assay, which reflects total antioxidant capacity, also highlighted the root extract (EC_50_ = 0.95 mg/mL) as comparably effective, suggesting the presence of redox-active non-phenolic components.

The ferrous ion chelating (FIC) test, used to estimate metal-binding capacity, revealed a distinct trend compared to the other systems: the stem extract exhibited the highest chelating activity (IC_50_ = 1.92 mg/mL), while flowers were markedly less efficient (IC_50_ = 6.06 mg/mL). This divergence indicates that metal-binding compounds in *H. pestalozzae* do not necessarily coincide with its radical-scavenging constituents.

**Figure 3:**
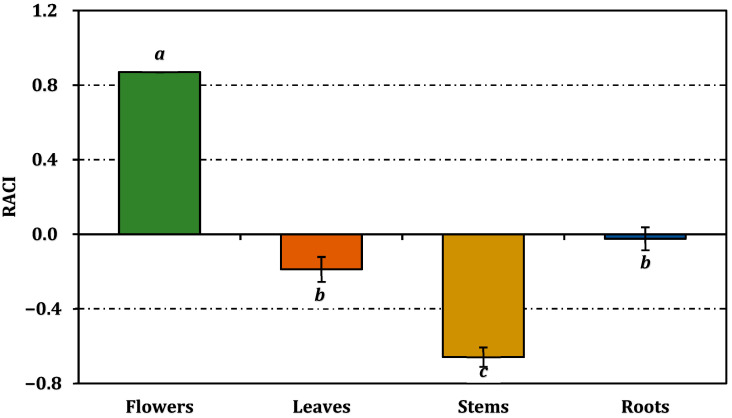
RACI of *H. pestalozzae* extracts. Values indicated by the same superscripts (a–c) on the bar chart are not significantly different according to Tukey’s HSD test at the 5% significance level.

**Figure 4:**
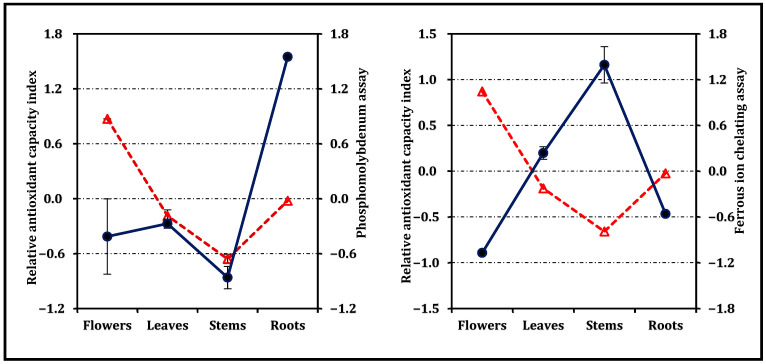

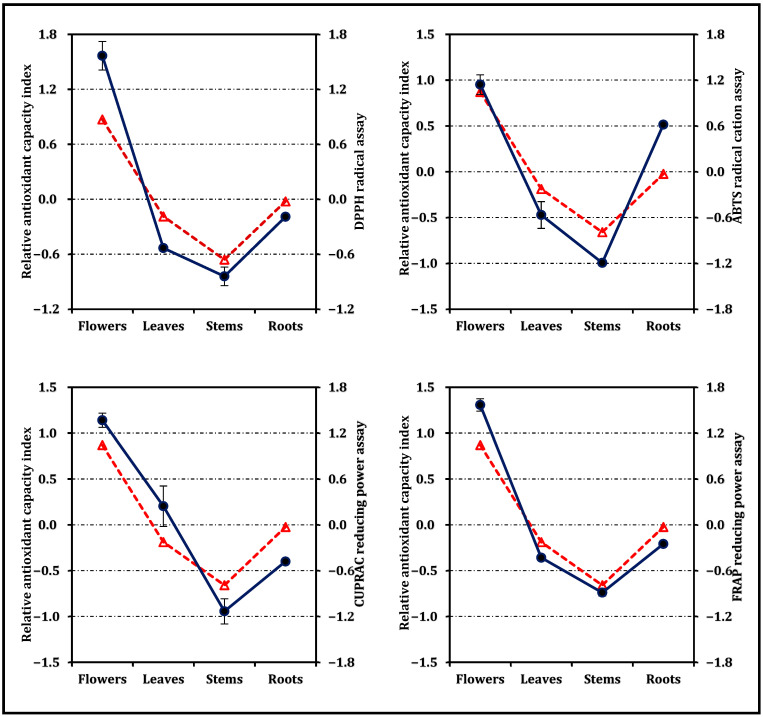
Correlation between the RACI (dashed red line with triangle) and antioxidant activity (solid dark blue line with circle).

To provide an integrated comparison of antioxidant responses, RACI values were calculated (Figure 3). The ranking of extracts followed the order: flowers > roots > leaves > stems, with RACI scores of 0.87, −0.02, −0.19, and −0.66, respectively. As shown in Figure 4, RACI values exhibited strong positive correlations with most antioxidant assays, except for the ferrous ion chelating test, supporting the overall consistency of antioxidant trends among plant organs and emphasizing the overall antioxidant superiority of the flower extract.

This study provides the first comprehensive assessment of the antioxidant capacity of *H. pestalozzae*. Results obtained across multiple test systems (CUPRAC, FRAP, DPPH, ABTS, metal chelation) revealed significant differences in activity among plant organs. The flower extract exhibited the highest performance, particularly in reducing power and radical scavenging tests, while the stem extract excelled in the metal chelation test. This suggests that the underlying antioxidant mechanisms are component-dependent and that metal-chelating capacity may not be directly linked to phenolic compounds.

The antioxidant activities observed in *H. pestalozzae* are generally consistent with previous reports on other *Hedysarum* species, supporting the genus as a rich source of antioxidant metabolites. For example, polysaccharides isolated from *H. polybotrys* exhibited effective radical scavenging activity, while ononin from the same species protected neuronal cells against oxidative damage [26,27]. Similarly, aqueous extracts of *H. austrosibiricum* enhanced antioxidant defense systems in vivo [28]. However, unlike these studies, the present work reveals pronounced organ-specific differentiation, with flower extracts showing superior electron-transfer and radical scavenging capacities, whereas stem extracts exhibited stronger metal-chelating activity. These findings suggest that antioxidant mechanisms in *H. pestalozzae* may vary considerably among plant organs.

In a recent study, it was shown that the high total phenolic (481 mg GAE/g) and flavonoid (26.6 mg QE/g) contents of the *H. nitidum* methanol extract were directly related to CUPRAC, FRAP, and DPPH activities [29]. Similarly, in *H. aucheri*, the butanol extract with the highest phenolic/flavonoid content showed the highest CUPRAC and ABTS activities [30]. In *H. alpinum*, phenolic compounds, flavonols, and polysaccharides were determined to contribute to radical scavenging activity [31]. Collectively, these comparisons indicate that the relationship between phenolic abundance and antioxidant activity appears to be conserved across the genus. Nevertheless, the pronounced organ-dependent specialization observed in *H. pestalozzae*, particularly the divergence between redox activity and metal chelation, represents a distinctive feature not commonly emphasized in previous studies.

On the other hand, the stronger activity of the stem extract in the metal ion chelation test suggests that non-phenolic compounds—such as organic acids, polysaccharides, or low-molecular-weight ligands—may also contribute to the antioxidant capacity. This observation is consistent with previous studies supporting the parallel operation of different antioxidant mechanisms in *Hedysarum* species [32].

The use of RACI analysis allowed for a holistic evaluation of results obtained from different test systems and revealed a general order of “flower > root > leaf > stem.” This order is consistent with the activity distribution based on phenolic/flavonoid abundance in other *Hedysarum* species. Therefore, *H. pestalozzae* is a pharmacologically noteworthy species, owing to its phenolic richness and versatile versatile antioxidant capacity.

### 2.3. Enzyme Inhibitory Activity

**Figure 5:**
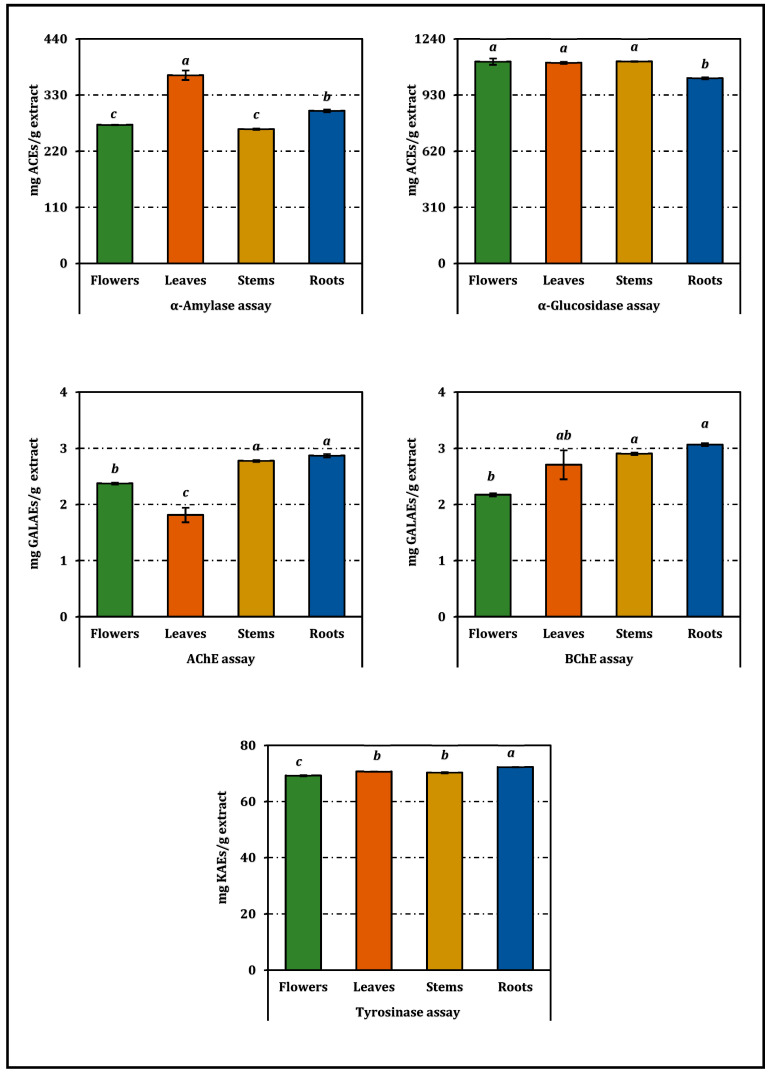
Enzyme inhibition activity of *H. pestalozzae* extracts. ACEs, GALAEs and KAEs mean acarbose, galanthamine and kojic acid equivalents, respectively. Values indicated by the same superscripts (a–c) on the bar chart are not significantly different according to Tukey’s HSD test at the 5% significance level.

The enzyme inhibitory potential of *H. pestalozzae* extracts was evaluated against key enzymes associated with neurodegenerative and metabolic disorders, including acetylcholinesterase (AChE), butyrylcholinesterase (BChE), tyrosinase, α-amylase, and α-glucosidase. The corresponding IC_50_ values are presented in Table 3 and illustrated in Figure 5.

All extracts exhibited moderate inhibitory effects on both cholinesterases, with AChE inhibition ranging from 1.11 to 1.77 mg/mL and BChE inhibition from 1.04 to 1.47 mg/mL. Among the tested parts, the root extract showed the strongest inhibition for both enzymes, followed closely by the stem extract. Although these values were markedly weaker than those of galanthamine (IC_50_ ≈ 0.003 mg/mL), the results indicate the presence of secondary metabolites capable of interacting with the catalytic sites of cholinesterases.

Regarding tyrosinase inhibition, the root extract showed the strongest activity (IC_50_ = 1.14 mg/mL), closely followed by the flower and other aerial extracts (IC_50_ ≈ 1.17–1.19 mg/mL), indicating comparable potency among organs. All samples were considerably less active than kojic acid (IC_50_ = 0.083 mg/mL), yet their consistent inhibition profiles suggest that phenolic derivatives, possibly flavonoids or phenylpropanoids, contribute to this effect.

In carbohydrate-hydrolyzing enzyme assays, the leaf extract displayed the most pronounced α-amylase inhibition (IC_50_ = 2.55 mg/mL), while the others were relatively weaker. Conversely, all extracts exhibited similar α-glucosidase inhibition profiles (IC_50_ ≈ 1.02–1.11 mg/mL), showing comparable potency to acarbose (IC_50_ = 1.14 mg/mL). These findings collectively suggest that *H. pestalozzae*, particularly its root and leaf extracts, contains compounds worthy of further investigation for enzyme-modulating properties relevant to neuroprotective and antidiabetic research.

The enzyme inhibitory profile of *H. pestalozzae* revealed a moderate yet functionally relevant bioactivity spectrum against cholinesterases, tyrosinase, and carbohydrate-hydrolyzing enzymes. Although the potency of its extracts was lower than that of the respective reference inhibitors, the observed inhibition patterns underscore the phytochemical richness of the species and suggest the presence of multiple classes of secondary metabolites capable of interacting with diverse enzymatic targets. Given that this is the first report on the enzyme inhibitory potential of *H. pestalozzae*, comparative assessment with other *Hedysarum* species provides important insights into its possible bioactive mechanisms.

In earlier studies, *H. nitidum* extracts demonstrated noteworthy inhibition of both cholinesterases and carbohydrate-hydrolyzing enzymes, particularly in their aqueous and methanolic fractions [29]. The moderate AChE and BChE inhibitory activities recorded for *H. pestalozzae* are in line with these findings, implying a shared phytochemical basis within the genus. Comparable results have been reported for *H. candidissimum*, in which methanolic extracts demonstrated pronounced AChE inhibitory activity, while molecular docking analyses suggested that phenolic acids and flavonoids are among the major contributors to this effect [33]. These structural classes are well known to form hydrogen bonds and π–π interactions within the active sites of cholinesterases, rationalizing the comparable inhibitory trends observed in *H. pestalozzae*. Although these observations are broadly consistent with previous reports on *Hedysarum* species, the present study additionally demonstrates clear organ-specific variation in enzyme inhibition profiles. In particular, root extracts exhibited stronger cholinesterase inhibition, whereas leaf extracts showed superior α-amylase inhibition, indicating differential distribution of enzyme-modulating constituents among plant organs.

The tyrosinase inhibition displayed by *H. pestalozzae* also parallels that of *H. nitidum*, whose extracts showed IC_50_ values in a similar range for both water and methanol fractions [29]. Since tyrosinase inhibition is often attributed to the presence of ortho-dihydroxylated phenolics and flavonols, the phenolic profile of *H. pestalozzae*—particularly rich in flavonoid constituents, as revealed by its chemical composition—likely contributes to this effect. Comparable inhibition patterns have been described in other *Hedysarum* taxa, including *H. varium* [34], supporting the genus-level potential for melanogenesis-related enzyme modulation.

Regarding the inhibition of α-amylase and α-glucosidase, *H. pestalozzae* demonstrated a balanced activity, with leaf extracts showing the most pronounced amylase inhibition and root extracts being particularly effective against α-glucosidase. These results mirror previous reports on *H. pallidum*, where butanol fractions exhibited strong α-glucosidase inhibition—remarkably surpassing that of acarbose [35]. Together, these comparisons suggest that the genus *Hedysarum* represents a promising source of antidiabetic agents targeting postprandial hyperglycemia through multiple enzyme inhibition routes. The inhibitory tendencies observed in *H. pestalozzae* generally align with previous findings in the genus; however, the organ-specific distribution of activities revealed here provides additional insight into the spatial organization of bioactive metabolites within the species.

Overall, the moderate-to-strong inhibition across all tested enzymes points toward a polyphenol-driven mechanism, consistent with the established correlation between phenolic content and enzyme inhibition in related species. Although quercetin derivatives, including hyperoside, have previously been reported to exhibit enzyme inhibitory activities, their high abundance in flower extracts did not consistently correspond to the strongest enzyme inhibition observed in the present study. This discrepancy may reflect the complex nature of crude plant extracts, where biological activity is influenced not only by the concentration of individual compounds but also by synergistic or antagonistic interactions among multiple constituents, differences in compound bioavailability, and varying affinities toward specific enzyme targets [36,37]. Therefore, enzyme inhibitory activities should be interpreted as the net outcome of the entire phytochemical matrix rather than the effect of single metabolites alone [36,38]. The observed enzyme inhibitory activities provide preliminary evidence supporting further studies on the pharmacological potential of *H. pestalozzae*, including fractionation-guided isolation, cellular assays, molecular docking, and in vivo validation. As the present findings are based on crude methanolic extracts and in vitro assays, definitive conclusions regarding therapeutic or nutraceutical applicability cannot yet be established. Future studies involving bioassay-guided fractionation, compound isolation, cellular assays, mechanistic investigations, and in vivo validation are necessary to confirm the biological significance and translational relevance of these observations.

It should be noted that the present findings are specific to methanolic extracts, and different extraction solvents may yield distinct phytochemical compositions and bioactivity profiles. In particular, food-grade solvents such as aqueous ethanol could provide extracts with different chemical characteristics and improved translational relevance for nutraceutical applications [39].

### 2.4. Correlations Among Phenolic Compounds and Assays

Pearson’s correlation analysis revealed exploratory associations between the phenolic composition of *H. pestalozzae* extracts and their bioactivity profiles (Table 4). In general, total phenolic and flavonoid contents exhibited significant positive correlations with most antioxidant assays, particularly FRAP (*r* = 0.883 and 0.903, respectively), CUPRAC (*r* = 0.945 and 0.825), and DPPH (*r* = 0.858 and 0.893). These findings confirm that the reducing and radical scavenging abilities of the extracts are largely driven by their phenolic richness. In contrast, the ferrous ion chelating assay showed strong negative correlations with these parameters, suggesting that metal-chelating activity depends on distinct non-phenolic constituents.

Among individual compounds, hesperidin, quercetin, syringic acid, and hyperoside were the most influential contributors to antioxidant potential, showing consistently high correlations with both electron-transfer assays (FRAP, CUPRAC) and the composite RACI index (*r* > 0.90). Conversely, verbascoside and (+)-catechin correlated negatively with radical scavenging and reducing assays but positively with FICA, supporting their preferential role in metal ion sequestration.

The cholinesterase inhibitory assays demonstrated moderate to strong negative correlations with total phenolics and most flavonoids, particularly with hyperoside (*r* = −0.963) and quercetin (*r* = −0.945), indicating that higher antioxidant capacity does not necessarily coincide with stronger enzyme inhibition. Tyrosinase inhibition showed a similar inverse trend with reducing assays but was positively correlated with vanillin (*r* = 0.934) and eriodictyol (*r* = 0.756), suggesting that certain minor phenolics may selectively contribute to this activity.

Regarding carbohydrate-hydrolyzing enzymes, α-glucosidase inhibition was positively associated with several hydroxybenzoic and hydroxycinnamic acids, such as *p*-coumaric, protocatechuic, gallic, 3-/4-hydroxybenzoic and ferulic acids, while α-amylase inhibition correlated weakly with the general antioxidant parameters. However, a relatively high positive correlation was found between ferulic acid, sinapic acid and taxifolin and α-amylase inhibition. Overall, the correlation patterns illustrated in Table 4 indicate that distinct subsets of phenolic compounds in *H. pestalozzae* differentially modulate antioxidant and enzyme inhibitory responses, underscoring the multifaceted chemical basis of its bioactivity profile. To facilitate visualization of the multidimensional relationships among phytochemical constituents and biological activities, a Pearson correlation heatmap was generated and is provided as Supplementary Figure S1. The heatmap highlights clusters of compounds and bioactivities exhibiting similar correlation patterns, thereby supporting the integrative interpretation of organ-specific chemical and functional profiles. Given the limited number of biological categories analyzed (*n* = 4 plant organs), the present correlation analyses should be interpreted as exploratory rather than confirmatory and are intended to identify potential associations requiring further validation in larger datasets. Because RACI is mathematically derived from individual antioxidant assays, correlations involving RACI should be interpreted cautiously and primarily as indicators of overall antioxidant trends rather than independent biological relationships.

## 3. Materials and Methods

### 3.1. Plant Material

*H. pestalozzae* was collected during its flowering stage on 1 August 2025, from gypsum-rich areas of Kümbet village in the Zara district of Sivas, Türkiye (39°48′23″ N, 37°48′44″ E; 1510 m altitude). The species identification was performed by Dr. Bedrettin Selvi, and a voucher specimen (GOPU 9615) was deposited in the Herbarium of the Faculty of Arts and Sciences, Tokat Gaziosmanpaşa University. Four distinct plant parts—flowers, leaves, stems, and roots—were individually collected during harvesting. The materials were air-dried in a shaded, well-ventilated area for several weeks and subsequently pulverized into fine powder using a laboratory grinder before analysis.

### 3.2. Methanol Extraction

All solvents, chemicals, and reagents used throughout this study were of analytical or LC–MS grade unless stated otherwise. HPLC-grade methanol, the gallic acid and rutin employed as calibration standards, the phenolic reference substances listed in the Supplementary File, together with the reagents required for the total phenolic, total flavonoid, antioxidant, and enzyme inhibition assays, were obtained from Sigma-Aldrich (St. Louis, MO, USA) and Fluka (St. Louis, MO, USA). Reference standards not available from these two suppliers were purchased from HWI Analytik (Ruelzheim, Germany). LC–MS grade formic acid was supplied by Merck (Darmstadt, Germany), while ultra-pure water was produced in-house using a Milli-Q Plus purification system (Millipore, Bedford, MA, USA).

After drying under shaded and low-humidity conditions, the plant samples were separated into their respective organs: flowers, leaves, stems, and roots. Each portion was ground to a fine consistency using a mechanical blender. Ultrasonic-assisted extraction was performed with methanol for 1 h in a sonication bath (Elma Schmidbauer GmbH, Singen, Germany), maintaining a sample-to-solvent ratio of 1:20 [40,41]. The extracts were concentrated under reduced pressure using a rotary rotary evaporator (Heidolph Instruments, Schwabach, Germany) and kept at 4 °C until further analysis. The extraction yields for the methanol extracts of flowers, leaves, stems, and roots were 7.31%, 6.11%, 5.47%, and 3.96%, respectively. Methanol was selected as the extraction solvent because of its well-documented ability to recover phenolic compounds across a broad polarity range and its widespread use in phytochemical studies, thereby facilitating comparisons with previous investigations on *Hedysarum* species and related taxa [39].

### 3.3. Determination of the Phenolic Composition

Total phenolic and flavonoid contents of the methanolic extracts were determined spectrophotometrically on a Shimadzu UV–Vis spectrophotometer (Shimadzu Corporation, Kyoto, Japan) using a previously reported procedure [42,43,44,45]. The targeted qualitative and quantitative analysis of selected phenolic compounds was conducted using a previously validated LC-ESI-MS/MS method [46]. The analytical validation parameters for this method are provided in Tables S1 and S2 of the Supplementary File. Chromatographic separation and detection were performed on an Agilent Technologies 1260 Infinity liquid chromatography system coupled to a 6420 Triple Quadrupole mass spectrometer (Agilent Technologies, Santa Clara, CA, USA), equipped with a Poroshell 120 EC-C18 column (100 mm × 4.6 mm, 2.7 µm particle size; Agilent Technologies, Santa Clara, CA, USA). Data acquisition and quantification were carried out using Agilent MassHunter Workstation software (Agilent Technologies, Santa Clara, CA, USA). The LC–ESI–MS/MS method employed in this study represents a targeted analytical approach based on the quantification of predefined phenolic standards. Therefore, the obtained data reflect the distribution of selected phenolic constituents rather than the complete metabolomic composition of *H. pestalozzae*. More comprehensive phytochemical characterization would require untargeted metabolomic approaches [24]. Representative LC–ESI–MS/MS chromatograms of methanolic extracts from flowers, leaves, stems, and roots are provided in Supplementary Figure S2.

### 3.4. Biological Activity Assays

The antioxidant and enzyme inhibitory activities of the extracts were evaluated using a series of in vitro assays. Antioxidant capacity was determined by multiple complementary methods, including phosphomolybdenum, 2,2-diphenyl-1-picrylhydrazyl (DPPH) radical scavenging, 2,2′-azino-bis(3-ethylbenzothiazoline-6-sulfonic acid) (ABTS) cation radical scavenging, cupric reducing antioxidant capacity (CUPRAC), ferric reducing antioxidant power (FRAP), and metal chelating assays, as previously described [47,48,49,50]. Enzyme inhibitory activities were evaluated against α-amylase, α-glucosidase, tyrosinase, acetylcholinesterase (AChE), and butyrylcholinesterase (BChE) using established spectrophotometric methods [51,52]. Detailed experimental conditions for all biological assays are presented in the Supplementary File.

### 3.5. Statistical Analysis

Each assay was performed in technical triplicate (*n* = 3), and the results were expressed as mean ± standard deviation (SD). Prior to statistical analysis, normality of the data was assessed using the Shapiro–Wilk test, while homogeneity of variances was evaluated using Levene’s test. The data met the assumptions required for parametric analyses. Statistical significance was evaluated through one-way ANOVA followed by Tukey’s post-hoc test at a confidence level of *p* < 0.05, using SPSS v26.0. Pearson correlation coefficients were calculated to explore relationships between chemical composition and biological activities. Considering the distinct mechanisms underlying different antioxidant assays, direct comparisons were not appropriate. Therefore, the RACI was computed to normalize and integrate the antioxidant data across assays. RACI was employed as an integrative descriptive tool to facilitate comparison among antioxidant assays with different response scales rather than as an independent biological variable. RACI values were obtained by standardizing each dataset—subtracting the mean and dividing by the standard deviation—and were subsequently correlated with individual assay results to provide an overall assessment of antioxidant trends across plant organs [53].

## 4. Conclusions

This study provides the first organ-specific assessment of targeted phenolic composition and associated biological activities in *Hedysarum pestalozzae*. Distinct chemical phenotypes were revealed among the examined organs, highlighting flowers as the major repositories of flavonols and phenolic acids, leaves as sources rich in hydroxycinnamates, stems as reservoirs of catechin- and verbascoside-type compounds, and roots as unique accumulators of vanillin and eriodictyol. Such spatial distribution patterns may reflect organ-dependent differences in secondary metabolite accumulation, which may contribute to the observed differences in bioactivity.

Consistent with their compositional richness, the flower extracts exhibited the most potent antioxidant capacities across multiple assays, suggesting a potential contribution of flavonols such as hyperoside, quercetin, and kaempferol in electron transfer and radical neutralization. The divergence of ferrous ion chelation from other redox assays further indicated the contribution of distinct, non-flavonoid chelators to the plant’s metal-binding potential. Enzyme inhibition profiles demonstrated a broader pharmacological relevance: roots and stems were relatively more effective against cholinesterases, while leaves showed the strongest α-amylase inhibition, and all organs displayed comparable α-glucosidase suppression, suggesting a potential multitarget inhibitory profile relevant to neuroprotective and antidiabetic research.

Correlation analyses revealed associations between phenolic composition and biological activities. The strong positive associations of total phenolics and specific flavonoids with reducing assays suggested that these compounds may contribute substantially to antioxidant responses, whereas inverse or selective relationships with enzyme inhibition highlighted the functional diversity of these metabolites.

Altogether, *H. pestalozzae* exhibits organ-dependent chemical diversity and measurable biological activities, combining redox-active and enzyme-modulating components in an organ-dependent manner. These findings not only enrich the phytochemical understanding of the genus *Hedysarum* but also position *H. pestalozzae* as a promising candidate for future investigations into natural antioxidant and enzyme-inhibitory agents.

## Figures and Tables

**Table 1:** Concentration (µg/g extract) of selected phenolic compounds in *H. pestalozzae* extracts.

No	Compounds	Flowers	Leaves	Stems	Roots
1	Hyperoside	6025 ± 28 *^a^*	2750 ± 7 *^b^*	1376 ± 2 *^c^*	127 ± 1 *^d^*
2	Kaempferol	3436 ± 6 *^a^*	2671 ± 39 *^b^*	254 ± 6 *^c^*	nd
3	Protocatechuic acid	2183 ± 38 *^a^*	854 ± 15 *^b^*	479 ± 4 *^c^*	41.7 ± 0.6 *^d^*
4	Hesperidin	1946 ± 13 *^a^*	327 ± 2 *^b^*	60.8 ± 3.8 *^c^*	38.8 ± 0.9 *^c^*
5	Quercetin	1916 ± 18 *^a^*	509 ± 25 *^b^*	96.9 ± 0.6 *^c^*	41.4 ± 3.0 *^c^*
6	Syringic acid	417 ± 11 *^a^*	27.7 ± 0.9 *^c^*	31.3 ± 0.2 *^c^*	72.6 ± 0.9 *^b^*
7	Gallic acid	414 ± 13 *^a^*	193 ± 1 *^b^*	118 ± 2 *^c^*	12.8 ± 0.3 *^d^*
8	(+)-Catechin	313 ± 3 *^c^*	635 ± 12 *^b^*	1489 ± 30 *^a^*	185 ± 9 *^d^*
9	Luteolin	285 ± 9 *^a^*	216 ± 5 *^b^*	20.5 ± 0.1 *^c^*	2.35 ± 0.18 *^c^*
10	3-Hydroxybenzoic acid	246 ± 3 *^a^*	131 ± 1 *^b^*	96.5 ± 2.1 *^c^*	41.3 ± 2.0 *^d^*
11	4-Hydroxybenzoic acid	246 ± 3 *^a^*	127 ± 1 *^b^*	97.5 ± 2.3 *^c^*	39.0 ± 0.7 *^d^*
12	*p*-Coumaric acid	120 ± 1 *^b^*	152 ± 2 *^a^*	65.2 ± 2.8 *^c^*	26.3 ± 0.4 *^d^*
13	Ferulic acid	72.0 ± 0.7 *^b^*	330 ± 3 *^a^*	40.8 ± 3.7 *^c^*	32.4 ± 2.2 *^c^*
14	Verbascoside	67.3 ± 0.1 *^c^*	270 ± 2 *^b^*	339 ± 12 *^a^*	245 ± 3 *^b^*
15	Caffeic acid	21.9 ± 0.5 *^b^*	34.1 ± 0.1 *^a^*	9.76 ± 0.07 *^c^*	25.6 ± 2.1 *^b^*
16	Sinapic acid	11.4 ± 0.3 *^b^*	21.3 ± 0.2 *^a^*	7.38 ± 0.60 *^c^*	7.18 ± 0.71 *^c^*
17	Luteolin 7-glucoside	10.2 ± 0.1 *^b^*	2.39 ± 0.47 *^c^*	72.7 ± 2.7 *^a^*	7.07 ± 0.83 *^b^*
18	Vanillin	7.78 ± 0.04 *^d^*	17.3 ± 0.9 *^c^*	24.4 ± 1.1 *^b^*	68.1 ± 0.7 *^a^*
19	Taxifolin	7.36 ± 0.02 *^b^*	19.2 ± 0.1 *^a^*	nd	nd
20	Eriodictyol	4.78 ± 0.25 *^b^*	2.67 ± 0.05 *^c^*	2.49 ± 0.25 *^c^*	13.2 ± 0.5 *^a^*
21	Chlorogenic acid	4.19 ± 0.13 *^d^*	107 ± 1 *^a^*	48.8 ± 1.4 *^b^*	16.8 ± 0.3 *^c^*

Values indicated by the same superscripts (a–d) within the same row are not significantly different according to Tukey’s HSD test at the 5% significance level. nd: Not detected.

**Table 2:** Antioxidant activities of *H. pestalozzae* extracts.

Assays	Flowers	Leaves	Stems	Roots	Trolox	EDTA
Phosphomolybdenum (EC_50_: mg/mL)	1.40 ± 0.14 ^c^	1.35 ± 0.02 ^c^	1.56 ± 0.05 ^c^	0.95 ± 0.03 ^b^	0.46 ± 0.05 ^a^	-
CUPRAC reducing power (EC_50_: mg/mL)	1.14 ± 0.02 ^b^	1.46 ± 0.10 ^c^	2.21 ± 0.14 ^d^	1.77 ± 0.05 ^c^	0.18 ± 0.01 ^a^	-
FRAP reducing power (EC_50_: mg/mL)	0.45 ± 0.01 ^b^	0.85 ± 0.01 ^d^	1.07 ± 0.03 ^e^	0.78 ± 0.01 ^c^	0.052 ± 0.006 ^a^	-
DPPH radical (IC_50_: mg/mL)	3.26 ± 0.14 ^b^	7.47 ± 0.11 ^c^	9.26 ± 0.72 ^d^	6.17 ± 0.02 ^c^	0.27 ± 0.02 ^a^	-
ABTS radical cation (IC_50_: mg/mL)	1.65 ± 0.04 ^b^	2.47 ± 0.12 ^c^	3.01 ± 0.01 ^d^	1.84 ± 0.12 ^b^	0.18 ± 0.02 ^a^	-
Ferrous ion chelating (IC_50_: mg/mL)	6.06 ± 0.02 ^e^	2.82 ± 0.09 ^c^	1.92 ± 0.13 ^b^	4.20 ± 0.38 ^d^	-	0.019 ± 0.001 ^a^

EDTAEs mean ethylenediaminetetraacetic acid (disodium salt). Values indicated by the same superscripts (a–e) within the same row are not significantly different according to Tukey’s HSD test at the 5% significance level.

**Table 3:** Enzyme inhibition activity of *H. pestalozzae* extracts.

Samples	AChE Inhibition (IC_50_: mg/mL)	BChE Inhibition (IC_50_: mg/mL)	Tyrosinase Inhibition (IC_50_: mg/mL)	α-Amylase Inhibition (IC_50_: mg/mL)	α-Glucosidase Inhibition (IC_50_: mg/mL)
Flowers	1.35 ± 0.01 *^b^*	1.47 ± 0.02 *^c^*	1.19 ± 0.003 *^d^*	3.46 ± 0.01 *^d^*	1.02 ± 0.02 *^a^*
Leaves	1.77 ± 0.13 *^c^*	1.19 ± 0.11 *^b^*	1.17 ± 0.003 *^c^*	2.55 ± 0.07 *^b^*	1.02 ± 0.01 *^a^*
Stems	1.15 ± 0.01 *^b^*	1.10 ± 0.01 *^b^*	1.17 ± 0.003 *^c^*	3.57 ± 0.02 *^d^*	1.02 ± 0.01 *^a^*
Roots	1.11 ± 0.01 *^b^*	1.04 ± 0.01 *^b^*	1.14 ± 0.003 *^b^*	3.14 ± 0.03 *^c^*	1.11 ± 0.01 *^b^*
Galanthamine	0.0034 ± 0.0004 *^a^*	0.0033 ± 0.0003 *^a^*	-	-	-
Kojic acid	-	-	0.083 ± 0.003 *^a^*	-	-
Acarbose	-	-	-	0.95 ± 0.04 *^a^*	1.14 ± 0.02 *^b^*

Values indicated by the same superscripts (a–d) within the same column are not significantly different according to Tukey’s HSD test at the 5% significance level.

**Table 4:** Pearson correlation coefficients among total phenolic and flavonoid contents, phenolic compounds, antioxi-dant assays, and enzyme inhibitory activities across the investigated organs of *H. pestalozzae*.

	TAP	DPPH	ABTS	CUPRAC	FRAP	FICA	AChEIA	BChEIA	TIA	AAIA	AGIA
DPPH radical	−0.032										
ABTS radical cation	0.461	0.854									
CUPRAC reducing power	−0.118	0.874	0.721								
FRAP reducing power	−0.049	0.997	0.840	0.903							
Ferrous ion chelating	−0.482	−0.822	−0.952	−0.765	−0.824						
RACI	0.105	0.972	0.908	0.924	0.979	−0.898					
AChE inhibition	0.363	−0.087	0.087	−0.538	−0.152	0.095					
BChE inhibition	0.455	−0.823	−0.498	−0.888	−0.843	0.489	0.464				
Tyrosinase inhibition	0.837	−0.544	−0.051	−0.565	−0.564	0.018	0.353	0.816			
α-Amylase inhibition	0.151	−0.323	−0.189	0.119	−0.265	−0.028	−0.766	0.151	0.291		
α-Glucosidase inhibition	−0.929	0.124	−0.366	0.278	0.159	0.354	−0.500	−0.596	−0.866	−0.055	
Total flavonoid	−0.461	0.893	0.538	0.825	0.903	−0.508	−0.239	−0.936	−0.859	−0.355	0.548
Total phenolic	0.185	0.858	0.853	0.945	0.883	−0.915	−0.415	−0.714	−0.301	0.179	−0.012
Hyperoside	−0.515	0.825	0.457	0.886	0.852	−0.480	−0.484	−0.963	−0.872	−0.109	0.632
Kaempferol	−0.425	0.671	0.378	0.909	0.720	−0.482	−0.773	−0.877	−0.716	0.290	0.580
Protocatechuic acid	−0.493	0.851	0.487	0.878	0.874	−0.497	−0.419	−0.962	−0.869	−0.175	0.611
Hesperidin	−0.301	0.954	0.673	0.899	0.964	−0.658	−0.255	−0.931	−0.758	−0.279	0.412
Quercetin	−0.334	0.930	0.636	0.920	0.948	−0.641	−0.345	−0.945	−0.776	−0.196	0.458
Syringic acid	−0.152	0.982	0.768	0.832	0.979	−0.718	−0.047	−0.854	−0.649	−0.427	0.255
Gallic acid	−0.550	0.804	0.417	0.858	0.831	−0.437	−0.469	−0.960	−0.893	−0.137	0.671
(+)-Catechin	−0.682	−0.598	−0.873	−0.624	−0.605	0.941	0.134	0.253	−0.262	−0.259	0.551
Luteolin	−0.414	0.684	0.392	0.912	0.733	−0.496	−0.757	−0.878	−0.716	0.278	0.575
3-Hydroxybenzoic acid	−0.558	0.805	0.417	0.854	0.831	−0.429	−0.458	−0.958	−0.900	−0.151	0.672
4-Hydroxybenzoic acid	−0.565	0.805	0.411	0.844	0.830	−0.420	−0.440	−0.957	−0.907	−0.174	0.676
p-Coumaric acid	−0.588	0.265	−0.053	0.636	0.326	−0.094	−0.942	−0.656	−0.637	0.536	0.716
Ferulic acid	−0.228	−0.225	−0.290	0.255	−0.160	0.086	−0.937	−0.127	−0.076	0.918	0.332
Verbascoside	−0.037	−0.989	−0.875	−0.913	−0.994	0.876	0.173	0.817	0.487	0.201	−0.091
Caffeic acid	0.389	0.092	0.311	0.458	0.141	−0.487	−0.690	−0.093	0.279	0.871	−0.239
Sinapic acid	−0.271	−0.049	−0.153	0.420	0.018	−0.043	−0.978	−0.297	−0.201	0.866	0.400
Luteolin 7-glucoside	−0.522	−0.445	−0.670	−0.658	−0.477	0.810	0.490	0.274	−0.209	−0.597	0.365
Vanillin	0.886	−0.354	0.137	−0.526	−0.392	−0.097	0.618	0.760	0.934	−0.016	−0.948
Taxifolin	−0.282	0.043	−0.085	0.502	0.111	−0.113	−0.988	−0.372	−0.264	0.836	0.418
Eriodictyol	0.946	0.087	0.561	−0.118	0.050	−0.509	0.558	0.416	0.756	−0.098	−0.957
Chlorogenic acid	−0.315	−0.637	−0.697	−0.210	−0.584	0.534	−0.705	0.226	0.066	0.804	0.336

Data show the Pearson Correlation Coefficients between the parameters. TAP: total antioxidant activity by phosphomolybdenum method. AAIA, AGAI, AChEIA, BChEIA and TIA: α-amylase, α-glucosidase, acetylcholinesterase, butyrylcholinesterase and tyrosinase inhibition activities, respectively. ABTS and DPPH: ABTS and DPPH radical scavenging activities, respectively. CUPRAC and FRAP: CUPRAC and FRAP reducing power potential, respectively. FICA: Ferrous ion chelating activity. RACI: Relative antioxidant capacity index.

## Data Availability

The data generated and/or analysed during this study are available from the corresponding author upon reasonable request.

## Supplementary Materials

The following supporting information can be downloaded at: https://www.mdpi.com/article/10.3390/molecules31132371/s1.
